# Low Concentrations of Gold Nanoparticles as Electric Charge Carriers in Piezoelectric Cement-Based Materials

**DOI:** 10.3390/ma17030615

**Published:** 2024-01-27

**Authors:** Daniel A. Triana-Camacho, Oscar A. Mendoza Reales, Jorge H. Quintero-Orozco

**Affiliations:** 1 Escuela de Física, Universidad Industrial de Santander, Cra 27 Calle 9, Bucaramanga 680002, Colombia; jhquinte@uis.edu.co; 2 Civil Engeneering Program, Universidade de Federal do Rio de Janeiro, Av. Pedro Calmon 550 Cidade Universitária, Rio de Janeiro 21941-901, Brazil; oscar@coc.ufrj.br

**Keywords:** cement paste, electrical impedance spectroscopy, electromechanical coupling, gold nanoparticles, piezoelectric voltage parameter

## Abstract

Piezoelectric cement-based composites could serve to monitor the strain state of structural elements or act as self-powered materials in structural health monitoring (SHM) applications. The incorporation of piezoelectric materials as an active phase within cement matrices has presented a highly attractive avenue until today. However, their application is challenged by the low electrical conductivity of the hydrated cement matrix. Gold nanoparticles (Au NPs) possess substantial potential for elevating the free electrical charge within the matrix, increasing its electrical conductivity between the Au NPs and the cement matrix, thereby enhancing the piezoelectric response of the composite. In this sense, the objective of this study is to investigate the effects of incorporating low concentrations of gold nanoparticles (Au NPs) (442 and 658 ppm) on the electrical and piezoelectric properties of cement-based composites. Additionally, this study considers the effects of such properties when the material is cured under a constant electric field. Electrical impedance spectroscopy was used to evaluate the polarization resistance and piezoresistive properties of the material. Additionally, open-circuit potential measurements were taken alongside the application of mechanical loads to assess the piezoelectric activity of the composites. The findings revealed a notable decrease in the composite’s total electrical resistance, reaching a value of 1.5 ± 0.2 kΩ, almost four times lower than the reference specimens. In the realm of piezoelectricity, the piezoelectric voltage parameter $g_{33}$ exhibited a remarkable advancement, improving by a factor of 57 when compared to reference specimens. This significant enhancement can be attributed to both the concentration of Au NPs and the electrical curing process. In summary, the outcomes of this study underscore the feasibility of creating a highly electrically conductive cement-based matrix, using low concentrations of gold nanoparticles as electric charge carries, and indicate the possible piezoelectric behavior of the studied compposite.

## 1. Introduction

The inclusion of nanomaterials in cement-based composites has been widely studied over the last two decades [1]. It is now known that nanoparticles such as carbon nanotubes (CNTs), amorphous silicon oxide, graphene oxide, aluminum oxide, iron oxide, and titanium oxide can modify the hydration reaction, rheology, mechanical properties, and durability of cement-based matrices [2,3]. Additionally, some of these nanomaterials can confer novel capabilities to cement-based composites such as self-sensing, self-cleaning, self-heating, electromagnetic shielding, and energy harvesting [4,5,6].

Among these novel capabilities, great potential has been found in the use of piezoelectric cement-based composites to develop energy-harvesting floors capable of generating a portion of the electricity consumed by a building [7]. Furthermore, piezoelectric properties extend beyond energy harvesting; these composites can generate energy without relying on external power sources to detect electric responses resulting from strain measurements, as observed in piezoresistive cement-based composites [8,9]. In other words, these nanocomposites have the ability to produce a small amount of voltage, electrical current, or power in response to applied strain, as demonstrated by Triana-Camacho et al. in their study on reduced graphene oxide–cement composites [10]. They also suggested that electric power could be a great candidate as a sensing parameter in real SHM applications. In this context, two parameters (the piezoelectric voltage parameter $g_{33}$ and the piezoelectric charge parameter $d_{33}$) play an important role in the characterization of the material’s piezoelectricity. The coefficient $g_{33}$ reveals how much electrical charge can polarize and generate a voltage in the cement-based composite, while $d_{33}$ shows how long it can preserve such polarization. In that sense, energy harvesting depends on both characteristics described above, which can be modified with different dispersion methodologies [10] or using different concentrations of the nanocomposite with respect to the cement mass [11]. Hence, the inherent self-sensing capabilities of piezoelectric cement-based composites can be effectively utilized in civil structures, including bridges [12], obviating the need for intricate monitoring systems or external transducers [13]. These embedded monitoring systems aim to identify and measure any degradation that might occur during service, avoiding degradation caused by weather changes or environmental conditions [12].

As a central concern, cement paste has minimal inherent piezoelectric behavior; moreover, the electrical current generated by mechanical stress constantly decreases as a function of cyclic loading [7]. Thus, the largest electrical current amplitude is observed during the first cycle of sinusoidal stress, followed by an intensity decrease in further stress cycles. These weak electrical phenomena can be explained by the water distribution in the cement structure, which causes electrical charge redistribution since cement paste is not a completely crystalline material [14]. Nonetheless, such inherent piezoelectricity of cement composites can be improved by applying an external electric field while curing cement-based composites, as shown by AlQaralleh [15], who improved the piezoelectricity of cement pastes while curing under an external voltage at 5 V, reaching up to three times the piezoelectric voltage parameter $g_{33}$ over nontreatment cement pastes. In addition, Ma [16] demonstrates that direct electric curing by 6 h makes cement obtain higher strength delaying ettringite formation.

Cement-based composites with piezoelectric materials have been developed with materials such as lead magnesium niobate ceramic [17], lead zirconate titanate (PZT)-silica fume [18], PZT-aluminum [19], and carbon black [20,21], as active phases. In addition, composites capable of generating higher amounts of electrical current have been considered appropriate for energy harvesting applications [7], while composites capable of generating lower amounts of electrical current have been considered appropriate for developing stress–strain sensors in smart civil infrastructure applications [22]. However, all of them exhibit a decline in electric current during subsequent load cycles, as Chen has declared in his review of piezoelectric cement-based composites [7].

Carbon nanotubes have been used as biosensors because they possess excellent electrical properties and a large specific surface area [23]. Nonetheless, not only are the electrical characteristics of the embedded nanomaterials important but so is the affinity with the host structure that allows for the identification of localized defects in the structure, as shown by Tung et al. in the reference [24] for graphene. For the same reasons, the electrical properties of Au NPs were also extrapolated to the cement industry to improve the electric or piezoelectric capability of cement composites [25]. A similar effect is expected to be achieved by increasing the number of electric charge carriers by incorporating metallic nanoparticles into the cementitious matrix. These metallic nanoparticles can be synthesized from a noble metal, known for its inherent resistance to corrosion effects. Considering the above limitations, and after a careful literature survey of the noble metals added to cementitious materials, it was found that gold nanoparticles (Au NPs) have not been studied for developing piezoelectric cement-based composites, except for the manuscript of Triana-Camacho and coauthors [26], who present the methodology to synthesize Au NPs, which are subsequently added to the cement paste. Interestingly, Au NPs have been commonly used in biosensor platforms or biomarker composites [27,28] and used to enhance the piezoelectric properties of polymer-based composites [29].

In this context, this work explores the use of Au NPs as a strategy to increase the free electrical charge in the cement paste and provide a piezoelectric response. As another novelty of this research, these metallic nanoparticles can also improve the piezoelectric response of nanocomposites already known as highly piezoelectric, such as PZT. Moreover, the electric properties in alternating current (AC) were also studied through electrical impedance spectroscopy to interpret the piezoresistive effects across polarization resistance.

## 2. Materials and Methods

The materials employed in this study include a gold plate measuring 30 mm in diameter and boasting a thickness of 0.15 mm. The gold plate, sourced from Kurt Lesker Company, in the United Kingdom, exhibited a purity of 99.9999%. Additionally, ultra-pure water derived from a Milli-Q IQ 7000 apparatus with a resistivity of 18.2 MΩ was utilized. Additionally, general-purpose Portland cement sourced from ARGOS-Colombia and a copper desoldering wire Hi-tronic manufactured in China, which measures 2.5 mm in diameter, was also used.

### 2.1. Gold Nanoparticles Synthesis and Characterization

Au NPs were produced by pulsed laser ablation in liquid (PLAL) with two different concentrations: low and high. The parameters used for the physical synthesis of gold nanoparticles were: temperature 25 °C, laser wavelength 530 nm, laser spot 12.6 ${\mathrm{mm}}^{2}$, the time between pulses $10^{-1}$ s, pulse duration 8 ns, and pulse energy 350 mJ [30]. The solvent (ultrapure water) volume that affects the optical path was 10 mL for the low-concentration Au NPs (442 ppm), and the ablation time was 5 min. For the high-concentration Au NPs batch (658 ppm), the solvent volume was 50 mL, and the ablation time was 10 min. The experimental setup mentioned above was selected considering the following criteria: (i) the total ablation time and the water volume has an impact on the size and concentration of the gold nanoparticles (Au NPs), as shown by Hernández-Maya and coauthors in the reference [30]. Moreover, (ii) the size of nanoparticles affects how these interact with the cement matrix [31]. Therefore, based on the previous statements, we tried to produce gold nanoparticles with a spherical morphology and size under 1000 nm, so that these Au NPs were incrusted inside the micropores produced by the characteristic hydration process, which takes place in the cement matrix, as well as diminishing the adverse effects on the mechanical properties on the cement-based composite [32]. In this sense, the concentrations used were the consequence of searching for the best conditions to produce gold nanoparticles of low sizes and proper geometry, as reported previously by Arevalo and coauthors [33].

The Au NPs particle size distribution was measured by dynamic light scattering (DLS) at a 90° angle 10 min after the synthesis using a particle analyzer Litesizer 500 from Anton Paar (Graz, Austria) together with the particle sizing software Kalliope (version 2.8.3). Each sample was tested in triplicate, and each result was an average from 6 measurements. In addition, the visible light absorbance of the obtained particles spectra was measured using an Agilent 8453 UV-visible spectrometer equipped with a deuterium-discharge (from 5301 Stevens Creek Blvd, Santa Clara, CA, USA) and tungsten lamps in 1 mm optical path quartz cuvettes.

Another way to determine the nanometric nature of Au NPs was through scanning electron microscopy (SEM). The measurement was performed by way of the FEI QUANTA FEG 650 SEM manufacture by FEI company (5350 NE Dawson Creek Dr, Hillsboro, OR, USA) with an acceleration voltage of 25 kV. Also, this equipment uses detectors for images: Everhart Thornley ETD for secondary electrons (SE), and backscattered electron (BSE) type SSD for chemical analysis (energy dispersive spectroscopy (EDS). In particular, an EDAX APOLO X detector was used with a resolution of 126.1 eV (Mn Kα). The data from EDS measurements were processed by the software EDX Genesis (version 5.2). It provides semi-quantitative information about the chemical elements in the cement composite.

### 2.2. Au NPs–Cement Composites Fabrication

To avoid any agglomeration or precipitation effects, the Au NPs were used after a maximum resting time of one hour before any experiment carried out in this study. Cement paste was hand mixed with a water-to-cement ratio (w/c) of 0.47, and Au NPs were added in two absolute concentrations (442 and 658 ppm). These low concentrations of gold in the matrix were chosen to minimize the gold consumption since the material developed would become unfeasible if large amounts of Au NPs were necessary.

**Figure 1:**
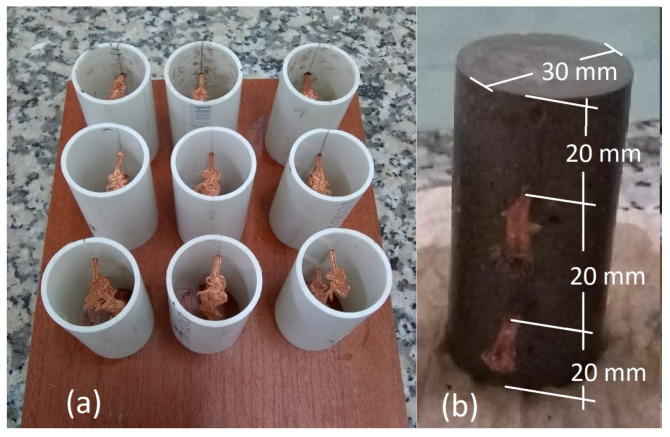
(**a**) Cylindrical molds with copper wire installed; (**b**) dimensions and configuration of each cylindrical sample.

A reference sample devoid of nanoparticles served as the control within the experiment. The precise composition of every paste is outlined in Table 1. The cement paste was cast into cylindrical molds measuring 60 mm in height and 30 mm in diameter. These molds were equipped with two holes, each measuring 3 mm in diameter, positioned at both 1/3 and 2/3 of the overall height. These apertures were utilized for embedding the copper wire into each sample, thereby functioning as measuring electrodes and maintaining a separation length of 20 mm. The configuration of the molds and the arrangement of the copper electrodes are illustrated in Figure 1, as was previously reported by Triana-Camacho et al. [26].

**Figure 2:**
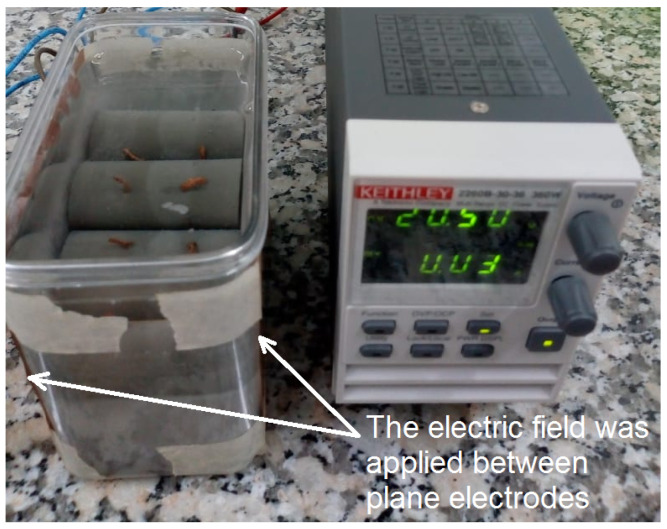
Curing of Au NPs–cement composite cylinders under an external electric field.

The cylinders were removed from the molds after 48 h of rest and then submerged in ultra-pure water for 28 days for curing. In total, 16 cylinders were made. The sample nomenclature was adopted according to their fabrication details, and the curing process are presented in Table 2 [26]. A group of 5 cylinders with different Au NP concentrations was cured and subjected to an external electric field to increase the cement paste’s inherent piezoelectric response [7]. The electric field was applied in the axial direction of the cylinders, between parallel copper plates, using a DC source at 20.5 V throughout the curing time. This curing setup is presented in Figure 2. At the end of the curing process, the cement paste cylinders were placed into an oven at 40 °C for 24 h to remove excess water in the pores of the composite [34].

The cylindrical specimens were extracted from the molds following a 48-h setting period and subsequently immersed in ultra-pure water for a curing duration of 28 days. Then, a total of 16 cylinders were produced. The nomenclature assigned to each sample was based on its fabrication particulars and the curing regimen, and this nomenclature is delineated in Table 2. A subset of 8 cylinders, each encompassing distinct Au NPs concentrations, was subjected to an external electric field (EF) to augment the inherent piezoelectric response of the cement paste. The application of the electric field was executed along the axial direction of the cylinders, positioned between parallel copper plates. A direct current (DC) source was employed, maintaining a consistent voltage of 20.5 V throughout the entire curing period [26]. This curing arrangement is depicted in Figure 2. Upon the culmination of the curing process, the cement paste cylinders underwent placement in a 40 °C oven for a span of 24 h, serving to eliminate excessive pore water within the composite [34].

### 2.3. Electric and Piezoelectric Characterization

**Figure 3:**
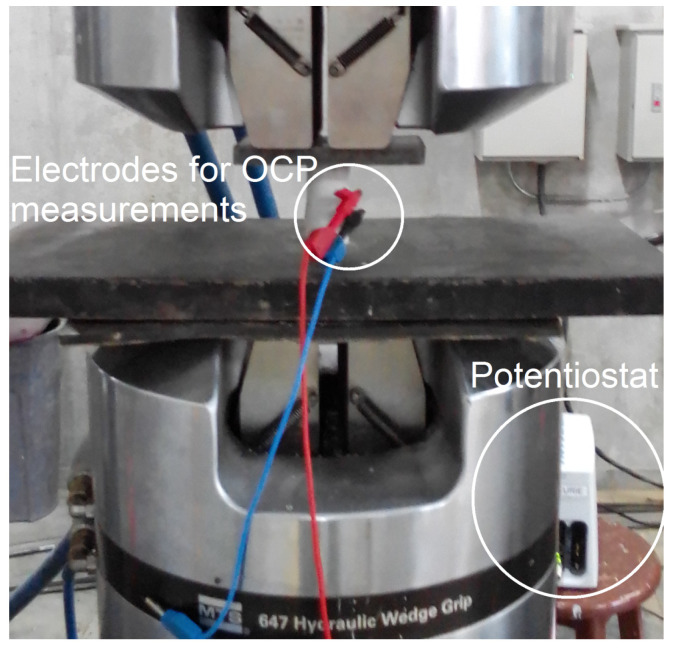
The experimental setup used to verify the piezoelectric properties of the Au NPs–cement composites.

Two sets of experiments were performed on the Au NPs–cement composites. First, their polarization electrical resistance was calculated based on electrical impedance spectroscopy (EIS) measurements, which was carried out using an AC potential signal of 10 mV while the frequency was swept from 1 MHz to 100 mHz in 60 evenly distributed points. Additionally, the specimens underwent extended EIS assessments spanning 253 days. This analysis aimed to track the temporal progression of both the polarization resistance and the heterogeneity parameter ϕ within the constant phase element (CPE), described by the model $Z_{CPE}=1/T{\left(j\omega \right)}^{\phi }$, where ω signifies angular frequency and T denotes pseudocapacitance [35]. Subsequently, for the assessment of their piezoelectric response, the specimens were testes in a universal testing machine (MTS-810 model, with a maximum force capacity of 500 kN). Here, a compressive load of 2.0 kN was applied in the axial direction, maintaining a constant loading rate of 0.02 kN/s. Simultaneously with the application of the load, the open-circuit potential (OCP) of each specimen was recorded. Importantly, it is worth noting that during the OCP measurements, no external voltage was imposed. This measure was taken to ensure that all outcomes directly corresponded to piezoelectric effects. A depiction of the experimental setup utilized for compressive load and OCP measurements can be found in Figure 3. For both electrical impedance and OCP measurements, a potentiostat/galvanostat, specifically the Autolab model PGSTAT204, was employed.

Linear regression was used to establish the effect of the compressive stress on the OCP measurements, showing that the continuous deformation of a heterogeneous material causes preferent polarization on the inherently piezoelectric nanocomposites, molecules, or inclusions; this is described by the linear theory of the piezoelectricity [36]. The piezoelectric voltage can be measured directly instead of polarization. It is known that there is a linear relationship between the piezoelectric voltage (which here is denoted as OCP) and mechanical sinusoidal input for cement-based composites too, based on the constitutive equations of piezoelectricity [37]. Therefore, taking into account that the mechanical force and the electric polarization inside the sample change in the same time interval and that the force rate is slower than the internal changes of polarization (it does not consider a delay between both quantities), time was used to equalize OCP and mechanical load as presented in Equation (1).

(1) V=γF+β.

where *V* is the OCP or piezoelectric voltage in (V); *F* is the applied force in (kN); according to the linearity between the OCP and force, the parameter β is the initial OCP (i.e., at zero load); and the parameter γ is the OCP-force coefficient in (mV/kN). The OCP-force coefficient can be defined as $\gamma =m_{v}/m_{F}$, where $m_{v}$ is the measured OCP rate and $m_{F}$ is the applied force rate.

## 3. Results

This section presents and discusses the results obtained from the experimental campaign and is divided into the synthesized Au NPs characterization, the piezoelectric response study, and the electric impedance characterization of the Au NPs–cement composites.

### 3.1. Au NPs Characterization

**Figure 4:**
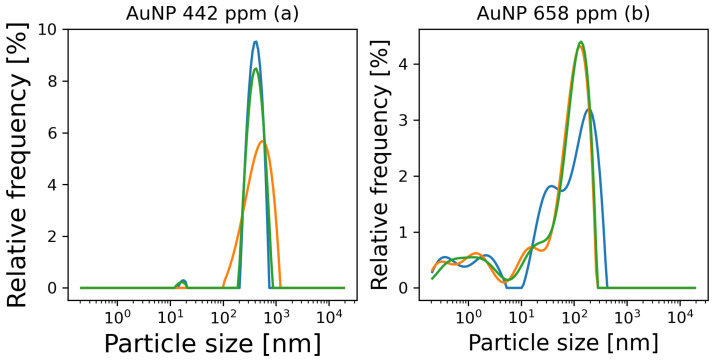
Particle size distribution results of the synthesized Au NPs in (**a**) low and (**b**) high concentrations.

The particle size distribution results obtained for the synthesized Au NPs at low and high concentrations are presented in Figure 4. It was found that the low concentration Au NPs (Figure 4a) had an average particle size $d_{50}$ of 407 ± 33 nm, with a bimodal size distribution with peaks at 17 nm and 450 nm. The high concentrations of Au NPs (Figure 4b) were found to have an average particle size $d_{50}$ of 77 ± 4 nm, with a trimodal size distribution with peaks at 2 nm, 25 nm, and 131 nm. These results indicate the nanometric nature of the particles synthesized in both concentrations.

**Figure 5:**
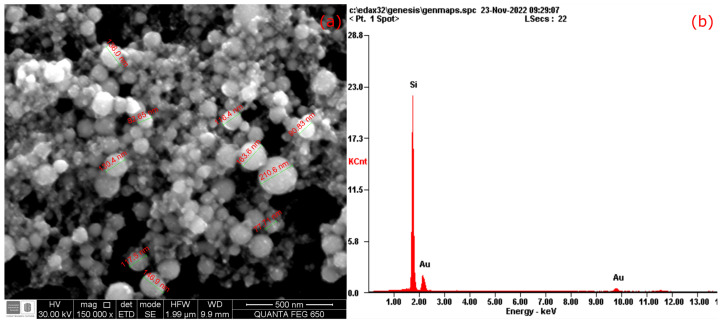
(**a**) SEM images and (**b**) EDS plots of gold nanoparticles for an ablation time of 10 min.

Moreover, the SEM images of Au NPs produce a more reliable estimation than DLS measurements of particle size, and the first was between 77.71 nm and 210.6 nm, as is observed in Figure 5a; in this setup, the Au NPs were obtained by an ablation time of 10 min, i.e., Au NPs were highly concentrated. Also, a chemical analysis was performed using Energy-Dispersive Spectroscopy (EDS) manufacture by FEI company (5350 NE Dawson Creek Dr, Hillsboro, OR, USA), which confirms the purity of Au NPs as illustrated in Figure 5b, and the spotlighted silicon peak was used for the wafer blank oriented at (110) crystal plane. In addition, the spherical shape of the Au NPs is also confirmed on the SEM micrograph because the PLAL can significantly affect both the morphology (producing elliptical Au NPs) and absorption traits [38,39]. The last comment is related to the fact that the Au NPs can form aggregates and affect their interaction with other components of the composites. Although this nanomaterial has not been added to a cement matrix, it is sure that the Au NPs sphericity will affect the rheological properties of the such cement-based composites.

**Figure 6:**
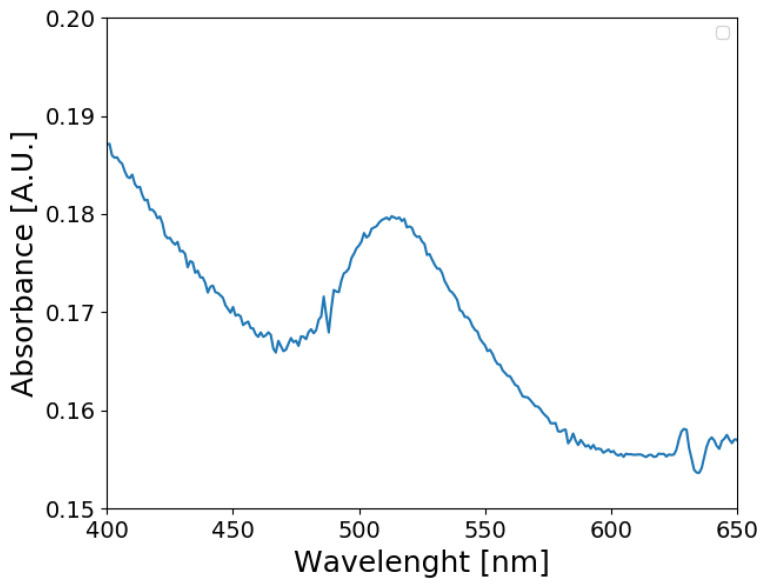
The visible light absorption spectrum of high-concentration synthesized Au NP.

On the other hand, a visible light absorption spectrum from the high-concentration Au NPs is presented in Figure 6. An absorption band was found between 475 and 550 nm of wavelength. This band can be related to the localized surface plasmon resonance, typical of gold nanoparticles as presented Li et al. [40], confirming the nature of the particles synthesized.

### 3.2. Piezoelectric Response

**Figure 7:**
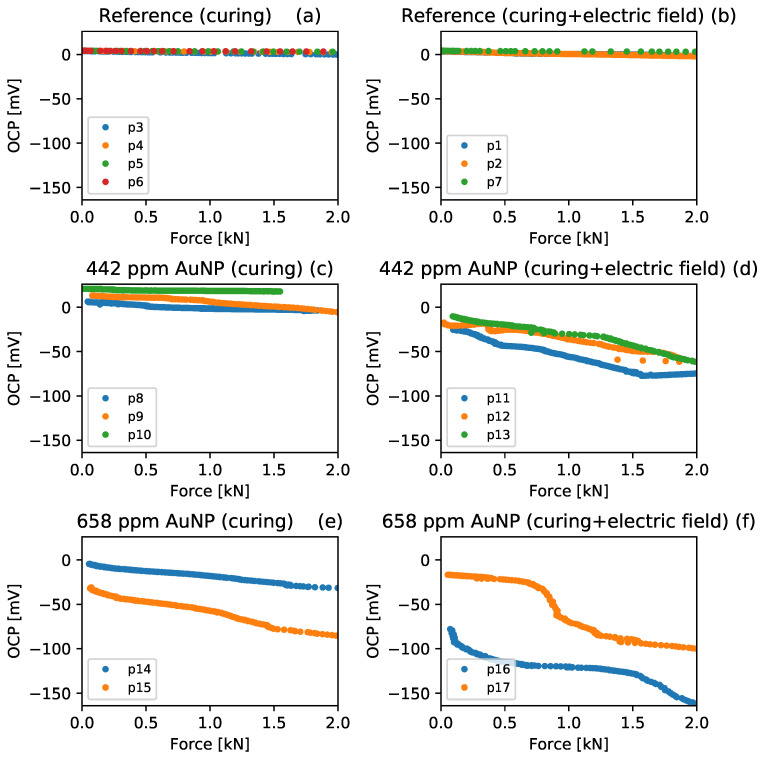
OCP results obtained from the piezoelectric tests of the Au NPs–cement composites studied: (**a**,**b**) reference specimens cured without and with an external electric field; (**c**,**d**) 442 ppm Au NPs specimens cured without and with an external electric field; and (**e**,**f**) 658 ppm Au NPs specimens cured without and with an external electric field.

The OCP variation with the load application for each studied sample and curing conditions is presented in Figure 7a–f. The OCP obtained from specimens without gold nanoparticles, which is presented in part (a) of Figure 7, presented an average 2.0 ± 1.3 mV potential difference between the initial and final mechanical loading states. This indicates the presence of piezoelectric properties in the cement paste, as suggested by Sun et al. [41]. Nevertheless, the electrical charges are not stable enough to sustain an electrical current or a potential difference for many charge cycles. Moreover, a 4.4 ± 2.2 mV variation in the results was obtained for the reference specimens cured under a constant electric field, as shown in part (b) of Figure 7, indicating that the presence of the electric field has an effect on the intrinsic piezoelectric response of pure cement paste. In addition, the OPC results obtained from specimens containing 442 ppm Au NPs, cured without and with an external electric field, are presented in parts (c) and (d) of Figure 7. At the end of the mechanical load application, the specimens showed an average OPC variation of 22.3 ± 12.0 mV for the specimens cured in water and 47.2 ± 4.3 mV for the specimens cured under a constant electric field. It can be seen that both the Au NPs presence, as well as the application of an external electric field, increase the OPC variation, indicative of an increase in piezoelectric activity. Finally, the OPC results for specimens with 658 ppm Au NPs, which are presented in parts (e) and (f) of Figure 7, present similar trends to those obtained with 442 ppm Au NP, finding an average OPC variation of 76.8 ± 40.6 mV for the specimens cured in water and 83.5 ± 0.1 mV for the specimens cured under a constant electric field. It is possible to affirm that both the addition of gold nanoparticles and the presence of a constant electric field during curing directly influence the piezoelectric response of the composite. These results are in agreement with the literature reports on cement paste where the OCP was found in the millivolts range and show an increment of piezoelectric properties after the cement was cured with an external electric field [42].

To better visualize the effect of the variables studied on the piezoelectric response of the Au NPs–cement composites, Equation (1) was fitted to the OCP results presented in Figure 7. The results obtained for the OCP-mechanical coupling (γ) were multiplied by the area of the cylinder cement sample (*A*) and divided between the separation of electrodes (*L*) to obtain the piezoelectric voltage coefficient $g_{33}=\left(A/L\right)*\gamma$; the OPC at time zero (β) also was obtained by fitting, and both parameters are presented in Table 3. It can be seen that $g_{33}$ increases to more positive values proportionally to the number of Au NPs added to the composites.

This more pronounced slope of the OPC results indicates an increase in piezoelectric activity, and its dependence on Au NPs can be associated with the following phenomena: (i) the high number of free electrons in the conduction band of Au NPs; (ii) the formation of continuous electrically conductive paths through the Au NPs; and (iii) the formation of conductive paths through hydration products inside the cement paste, as suggested by Song et al. [43], due to the refinement of the matrix microstructure. The $g_{33}$ values were found to increase furthermore with the application of an external electric field during curing until 146 ± 45 [$10^{-5}$ mVm/N]. This fact can be related to changes in the nanoscale morphology of cement paste due to the polarization of hydration products [42] and to the possible polarization of the Au NPs, and such values are approximately 10 times greater than the cement pastes polarized at 5 V during the curing stage as reported by AlQaralleh [15]. In conclusion, a pronounced slope in OCP curves corresponds to the same effect that provides more piezoelectric activity. This is associated with the high amount of free electrons in the conduction band of Au NPs and with the changes in the nanoscale morphology of the cement matrix due to the polarization of hydration products, as explained by Yaphary in reference [42]. Both phenomena interact, polarizing the free charge carriers inside Au NPs, hence increasing the piezoelectric response. Finally, the β parameter was found to decrease primarily with the number of Au NPs and secondarily with the presence of the electric field, indicating that the initial OCP value depends mainly on the Au NPs concentration.

### 3.3. Electrical Impedance

**Figure 8:**
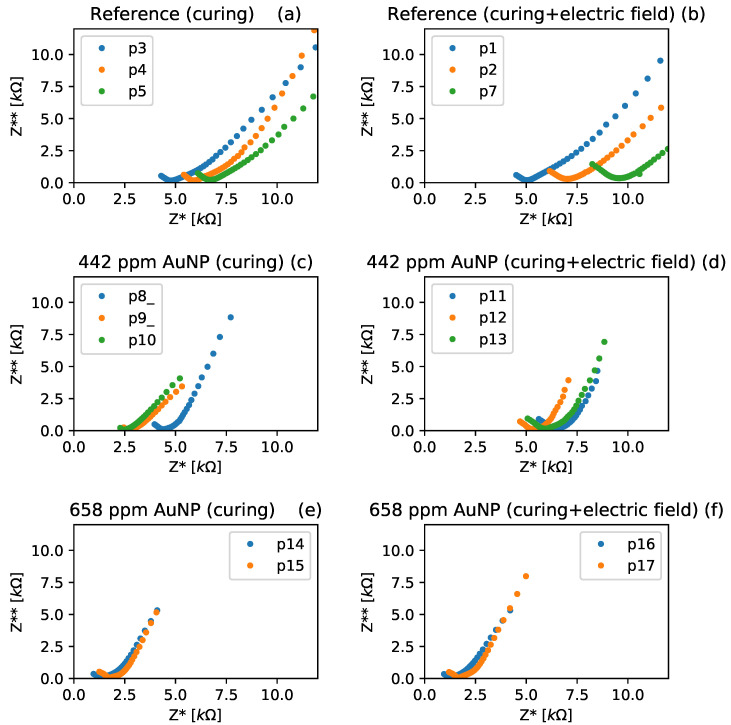
Electrical impedance results obtained for all the Au NPs–cement composites studied: (**a**,**b**) reference specimens cured without and with an external electric field; (**c**,**d**) 442 ppm Au NPs specimens cured without and with an external electric field; and (**e**,**f**) 658 ppm Au NPs specimens cured without and with an external electric field.

Nyquist curves for the Au NPs–cement composites studied are presented in Figure 8. The total resistance, i.e., the bulk resistance plus polarization resistance, can be obtained by fitting data of the complex and real parts of impedance to a semicircle in the Nyquist curves [44]. The semicircle corresponds to the Randles circuit model usually used to obtain the characteristic features of the concrete pore structure [45]. It consists of polarization resistance in parallel with internal capacitance, both in series with bulk resistance [46]. The outcomes derived from fitting real and complex impedance data to the Randles circuit model are displayed in Table 4. The acquired figures demonstrated a reduction in the overall resistance that corresponded to the concentration of Au NPs. This observation confirms the increase in electrically conductive paths within the specimens, aligning with the earlier discussions in this section.

On the other hand, the reference and 442 ppm Au NPs specimens cured under an electric field presented higher total resistance when compared to the specimens cured without it. From these results, it is clear that the curing under an electric field affects the total resistance of the Au NPs–cement composites. AlQaralleh [15] showed that curing Portland cement pastes under an electric field polarizes and aligns the hydrated nanostructures, improving the piezoelectricity of the material. This alignment may be related to a decrease in the electrical current paths by reducing the ion mobility [42], which increases the total material resistance. Further experiments would be necessary to confirm this hypothesis. The 658 ppm Au NP sample presented a slight decrease in its total resistance when cured under an electric field. This indicates that there is a concentration of Au NP above which the polarization effect has no negative effects over the total resistance of the composite because the electronic conductivity through the Au NP becomes more predominant than the ionic conduction through the hydrated nanostructures.

The total polarization resistance of three specimens (with different gold concentrations) was measured and labeled as “$R_{p}$ initial”. Then, 2 kN was applied to each specimen and maintained to measure the total polarization resistance after load application. Then, the result obtained was labeled as “$R_{p}$ final”. The obtained results are presented in Table 5.

It can be seen through the impedance curves that the cement-based specimens on gold nanoparticles have a frequency-dependent electrically conductive-resistive or dielectric behavior. In such a case, when the impedance has more resistive behavior, a state of polarization of the system that is called polarization resistance is accessed. In addition, this work showed the creation of an electric potential related to the deformation of the cement specimens with Au NPs. The potential is due to the polarization of the nanoparticles in the material. It can occur via the reorganization of the electric charges in the surface of the grains or via the rotation of the grains [47,48], being a solid embedded in a solid matrix; these effects are not considered with the translation of electric charges. Such polarization is accompanied by interactions between the dipole moments of neighboring grains. These forces decrease rapidly with distance; then, the possibility of electrical moving charges between dipoles is reduced, which would increase the resistivity of the matrix [49], as shown in Table 5. This result confirms that the matrix developed has no piezoresistive effects and that the use of FCR is inadequate for the characterization of cement composite based on gold nanoparticles.

**Figure 9:**
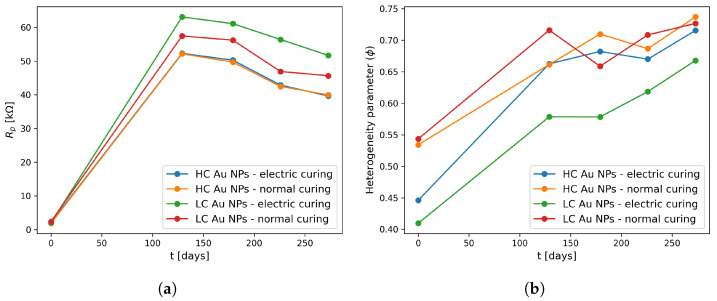
(**a**) Polarization resistance ($R_{p}$) and (**b**) heterogeneity parameter (ϕ) were derived from EIS measurements spanning 273 days. The specimens were classified into two categories: high concentration (HC) of Au NPs and low concentration (LC) of Au NPs. These specimens underwent treatment with an external electric field (electric curing) or without (normal curing).

On the other hand, the quasi-linear behavior at low frequencies in the Nyquist plots describes the compound’s interaction with the electrode. This interaction can be modeled using either a Warburg element [44] or a constant phase element (CPE) [48]. Consequently, upon incorporating nanoparticles into the cement (as depicted in Figure 8c–f), these linear elements exhibit steeper slopes in comparison to the reference specimens (refer to Figure 8a,b). Within the framework of CPE interpretation as Lasia et al. revised in their manuscript [50], these slopes are directly linked to the heterogeneity parameter ϕ. Therefore, the heightened slope implies more capacitive behavior due to the inclusion of Au NPs. Subsequent to this, the polarization resistance and the ϕ parameter were extracted from the Nyquist plots assessed for up to 273 days, as illustrated in Figure 9.

It is worth mentioning that regardless of the manufacturing configuration, the polarization resistance experiences growth as the specimens age up to 130 days. Subsequently, beyond this point, the electrical polarization resistance undergoes a slight decline, eventually stabilizing around 250 days, as depicted in Figure 9a. When comparing curves with equivalent concentrations, barring the variation in electrical curing, it can be deduced that the latter has a relatively minor impact on polarization resistance. However, an increase in concentration leads to a reduction in electrical resistance, as previously observed in the Nyquist curves within Figure 8. This infers that the nanoparticles have not interacted with the environment as anticipated for a noble metal, which is commonly used for designing electrochemical sensors [51]; instead, the cementitious matrix has experienced chemical reactions across the months, thereby leaving only the nanoparticles contributing to electrical conductivity. Furthermore, it is imperative to underscore that the practical application of these compounds within the realm of structural monitoring can only be fully comprehended over an extended period. The stabilization of polarization resistance suggests that these compounds could be deemed suitable for integration into real structures after an approximate 200-day duration.

The heterogeneity parameter defines how a circuital object called a CPE becomes a resistive, capacitive, or pseudocapacitive element, in order to model the electric behavior close to the electrode interface embedded in the cement-based composite. In that sense, the heterogeneity parameter (see Figure 9b) of all specimens exhibits a transition from an electrically resistive state towards more capacitive behavior. This transition is indicated by the fact that when ϕ reaches a value of 1, the interaction between the electrode and the cement composite with the addition of Au NPs becomes entirely capacitive. It is evident here that, as the nanoparticles may not be reacting on the electrode’s surface, it is the electrode itself that undergoes chemical changes by absorbing water from the cementitious matrix and forming an oxide layer, contributing to the overall capacitive behavior of the system, as occurs with steel embedded in cementitious materials [52].

Concerning the different sample configurations, the electrical curing does influence the pseudocapacitive behavior. Conversely, the nanoparticle concentration does not exert a significant effect on the ϕ parameter. This is because minimal differences are observed in the ϕ parameter between normally cured specimens with different concentrations (HC Au NPs and LC Au NPs) both initially (0 days) and at the final time point (273 days). Therefore, the normally cured specimens display more capacitive behavior at 273 days compared to the electrically cured specimens with varying concentrations (HC Au NPs and LC Au NPs). Drawing from the insights provided by [16], who subjected cement composites to electrical treatment resulting in improved C-H-S structure formation and reduced ettringite formation, it can be deduced that electrically cured cement composites with the addition of Au NPs also accelerated the hydration process. This acceleration led to a decrease in oxide formation on the electrode’s surface, represented by a less capacitive parameter value (around 0.65 at 273 days) compared to the normally cured specimens, which exhibited a value of around 0.73.

## 4. Conclusions

The inclusion of Au NPs in a cement-based composite was studied by means of electrical impedance spectroscopy to explore their use as an electrical charge carrier and to understand their effects on the piezoresistive response and polarization resistance of the composite. These properties were evaluated for 273 days to determine whether the electrical behavior can be maintained over a long period of time. It was found that the inclusion of a low concentration of Au NPs, such as 658 ppm, in a Portland cement matrix is capable of decreasing the total electrical resistance of the composite by almost four times and increases its piezoelectric response by 28 times. It was also observed that the use of an external electric field during the curing of the composites increased the piezoelectric response of the material by 57 times, proving to be an important step to be considered during the scaling-up process of a piezoelectric cement-based composite fabrication.

It should be highlighted that the increase identified in the $g_{33}$ could be also indicative of the piezoelectric behavior of the Au NPs. Further studies should be conducted to confirm this hypothesis. Additionally, further experiments could focus on combining low amounts of gold nanoparticles with known piezoelectric materials to identify if there is an increase in the efficiency of the piezoelectric effect of the cement-based composite. Regarding the price limitations of gold nanoparticles, it can be suggested that this composite is more suitable for the development of the small stress–strain embedded sensor and less suitable for the development of high-volume energy harvesting applications. Finally, to fully demonstrate the feasibility of Au NPs–cement composites, the effect of the nanoparticles on the chemical, mechanical, and durability properties of the hydrated cement matrix should be studied. Nevertheless, it is clear that the Au NPs confer a high number of electric carriers that can be used to enhance other piezoresistive or piezoelectric materials such as graphene, PZT, zeolites, and oxides, closing the gap to the real-world applications of piezoelectric cement-based composites. Examining the potential use of the developed composite as an embedded sensor within the same cylindrical geometry discussed in this research, and factoring in the current costs of gold nanoparticles, the investment required for producing a single sensor would range from USD 15 to 23. However, when combined with production costs and the expenses associated with gold recovery at the end of its life cycle, the commercialization of these composites becomes unfeasible for the proposed application, given the more economical alternatives available in the market. Nonetheless, the results showcased in this study underscore a promising application of metallic nanoparticles that could drive an uptick in industrial-scale production, subsequently leading to a reduction in prices—an anticipated trend in the current landscape of most nanomaterials.

As a final remark, it should be highlighted that, so far, there are no literature reports showing the recovery of any kind of nanomaterial from recycled concrete. Therefore, there are no clear schemes for recycling nanoparticles from solid cement-based composites. In the case of gold nanoparticles, one could use some chemical components with a high affinity with gold to absorb them from cement after a grinding process. Nevertheless, the chemical components commonly used in the synthesis of gold nanoparticles could be toxic and harmful to the environment, so it is necessary to evaluate the environmental impact of this process.

## Figures and Tables

**Table 1:** Au NPs–cement composites proportioning. (w/c: water-to-cement ratio).

Material	Reference	442 ppm Au NPs	658 ppm Au NPs
Cement (g)	60.0	60.0	60.0
Water (g)	28.2	28.2	28.2
Au NPs (mg)	-	39	58
w/c	0.47		

**Table 2:** Nomenclature of Au NPs–cement composites studied in this work.

Specimens	Normal Curing	Electrical Curing
Reference	p3, p4, p5, p6	p1, p2, p7
442 ppm Au NPs	p8, p9, p10	p11, p12, p13
658 ppm Au NPs	p14, p15	p16, p17

**Table 3:** Coupling OCP-mechanical coefficients obtained for the Au NPs–cement composites studied.

Sample	Normal Curing			Electrical Curing		
	$g_{33}$	β	$r^{2}$	$g_{33}$	β	$r^{2}$
	[$10^{-5}\frac{\mathrm{mVm}}{N}$]	[mV]		[$10^{-5}\frac{\mathrm{mVm}}{N}$]	[mV]	
Reference	2.5 ± 1.9	3.8 ± 0.2	0.99	6.6 ± 3.6	3.6 ± 0.3	0.98
442 ppm	18.3 ± 12.2	−12.9 ± 6.9	0.88	92.1 ± 12.3	−15.4 ± 7.0	0.97
658 ppm	72.4 ± 24.3	−18.9 ± 13.6	0.98	146.0 ± 45.0	−49.5 ± 44.6	0.88

**Table 4:** Total resistance obtained from electrical impedance results for the Au NPs–cement composites studied.

Sample	Total Resistance (kΩ)	
	Normal Curing	Electrical Curing
Reference	5.8 ± 0.8	7.2 ± 1.9
442 ppm Au NPs	3.2 ± 0.9	5.9 ± 0.5
658 ppm Au NPs	1.5 ± 0.2	1.4 ± 0.2

**Table 5:** Piezoresistive measurements, where fractional change in resistivity is presented as FCR.

Sample	$R_{p}$ Initial (Ω)	$R_{p}$ Final (Ω)	$\Delta R_{p}$ (Ω)	FCR%
Reference (electrical curing)	11,336.0	11,630.0	+294.0	2.6
442 ppm Au NPs (electrical curing)	10,968.6	11,269.1	+300.5	2.7
658 ppm Au NPs (electrical curing)	1563.5	1629.2	+65.7	4.2

## Data Availability

The research data and software can be found on GitHub repository https://github.com/dantrica/Au-NPs-cement-composites (accessed on 5 July 2023).

## Abbreviations

The following abbreviations are used in this manuscript:

SHM	Structural health monitoring
OCP	Open-circuit potential
Au NPs	Gold nanoparticles
FCR	Fractional change in resistance
CPE	Constant phase element
